# Dietary pattern is associated with obesity in Chinese children and adolescents: data from China Health and Nutrition Survey (CHNS)

**DOI:** 10.1186/s12937-018-0372-8

**Published:** 2018-07-11

**Authors:** Shihan Zhen, Yanan Ma, Zhongyi Zhao, Xuelian Yang, Deliang Wen

**Affiliations:** China Medical University, No.77 Puhe Road, Shenyang North New Area, Shenyang, Liaoning Province China

**Keywords:** Dietary patterns, Obesity, China, Adolescents, Children

## Abstract

**Background:**

Associations of dietary patterns in Chinese adolescents and children with later obesity have not previously been investigated. The purpose of the present study was to evaluate the associations between dietary patterns and the risk of obesity in Chinese adolescents and children by using a longitudinal design.

**Methods:**

Data from the China Health and Nutrition Survey (CHNS), a nationally representative survey, were used for our analysis. 489 participants 6–14 years of age were followed from 2006 to 2011. Factor analysis was used to identify the dietary patterns in Chinese adolescents and children. Ordered logistic regression models were used to examine the association between dietary patterns and later obesity.

**Results:**

Two dietary patterns were revealed by factor analysis, the traditional Chinese dietary pattern (with high intake of rice, vegetables, poultry, pork and fish and the modern dietary pattern (with high intake of wheat, processed meat and fast food). Children in the highest quartile and the second-highest quartile of the traditional Chinese dietary pattern was inversely associated with later obesity compared with children in the lowest quartile over 5 years (OR = 0.19, 95%CI: 0.09, 0.40 for Q4; OR = 0.47, 95%CI: 0.33, 0.67 for Q3); Children in the highest quartile of the modern dietary pattern was positively associated with later obesity compared with children in the lowest quartile over 5 years (OR = 2.02, 95%CI: 1.17, 3.48).

**Conclusions:**

Dietary patterns in Chinese adolescents and children are associated with later obesity. These findings further confirm the importance of children’s dietary patterns in later obesity and lay groundwork for dietary culture-specific interventions targeted at reducing rates of obesity in children and adolescents.

## Background

Childhood overweight and obesity have become a worldwide health problem. In developing countries, Childhood overweight and obesity have increased at an alarming rate [1, 2]. In 2014, about 35 million children were affected by overweight or obesity, and the prevalence of overweight and obesity for children in China was 12.2 and 7.3% respectively [1]. These large increases in the prevalence of childhood obesity might greatly increase morbidity in adulthood from causes such as cardiovascular disease, metabolic syndrome and diabetes [3–5]. Therefore, research identifying risk factors of childhood obesity are needed to address this severe public health problem.

Obesity is caused by an intricacy interaction among the environment, genetics and behavior [6]. Among these factors, diet has been demonstrated to be a determinant in the development of obesity [7]. However, owing to the complexity of diets and the potential associations between dietary components [8], the relationship between diet and obesity is intricate and barely understood [9]. Compared with traditional dietary analyses focusing on individual foods or nutrients [10], dietary pattern analysis might be an alternative holistic and comprehensive approach [11].

Dietary pattern analysis take advantage of complex diet, take into account multiple food groups instead of individual foods or nutrients, and can reveal potential interactions between various nutrients and food [11, 12]. Consequently, dietary pattern analysis might reflect the complexity of dietary intake and provide new insights into what people eat [13, 14]. Thus, dietary pattern analysis has widely been used to determine associations between diet and chronic diseases and to aid in formulating nutritional recommendations [15–17].

In China, transition of dietary patterns is an ongoing phenomenon [18]. Over the past 20 years, dietary patterns have transitioned from a traditional diet to a modern diet with high intake of refined grains and meat; the abundance of energy-dense food in the modern diet is cause for concern [13]. Dramatic changes in dietary patterns may explain the increase in obesity among children and adolescents [19]. However, to our knowledge, most previous studies have followed a cross-sectional design [15, 20] and have focused primarily on adults [21, 22]. Few studies have analyzed the long-term effects of dietary patterns and obesity in Chinese children or adolescents. Therefore, in the present study, we sought to characterize dietary patterns at baseline and to identify the associations between dietary patterns and later obesity in Chinese children and adolescents by using a longitudinal study design.

## Methods

### Study design and population

In the present investigation, we used longitudinal data from the China Health and Nutrition Survey (CHNS), an open prospective cohort study. CHNS used a multistage random-cluster sampling process to select samples from 15 provinces in China [23]. CHNS was approved by the Institutional Review Committees of the University of North Carolina at Chapel Hill and the National Institute of Nutrition and Food Safety, Chinese Center for Disease Control and Prevention. Details have been described elsewhere [24].

We used the longitudinal data from 2006 to 2011. A total of 736 participants aged 6–14 were involved in 2006. Among them, 489 participants completed all three surveys and were enrolled in the current analysis. We excluded participants with implausibly high or low caloric intakes (ie, < 600 or > 4000 kcal/d), missing dietary pattern data or anthropometry data, or those with a history of metabolic disease before baseline.

### Dietary assessment and food grouping

Details of dietary measurements have been provided elsewhere [25]. In brief, dietary were recorded through a 24-h-recall method for three consecutive days for each participant. For children younger than 12 years, parents were asked to recall children’s food consumption. Alcoholic beverages were excluded because of very low consumption in children.

Dietary were based on the food frequency questionnaire using in the survey in 2006. The dietary data were divided into 28 food groups (Table 1) based on their similarity in nutrients and Chinese Food Composition Table [26].

**Table 1:** Food groupings used in factor analysis

Food group	Examples of food items
Rice	White and brown rice
Wheat noodles	Wheat noodles
Wheat flour	Wheat flour
Wheat buns, breads	Bun, butter bread, salty bread
Cakes, cookies and pastries	Cookies, mooncake, fruit cake, chocolate cake, fruit pie
Deep-fried wheat	Deep-fried dough stick, deep-fried cake with red bean paste
Corn and coarse grain	Corn, corn grits, barley, oats, foxtail millet, sorghum
Starchy roots and tubers	Potato, yam, taro, lotus root, water chestnut, cassava
Fresh legumes	Soybean sprouts, peas with pod, mung bean sprouts
Dried legumes	Soybean flour, dried beans, beans flour, roasted broad bean
Legume products	Tofu, tofu products, red/mung bean paste
Nuts and seeds	Sesame, sunflower, lotus seeds, peanuts, walnuts, almonds, hazelnuts, pine-nuts, pistachios, cashew nuts
Fresh vegetables, non-leafy	Cauliflower, tomatoes, cucumber, zucchini, mushrooms
Fresh vegetables, leafy	Spinach, ‘bok choy’, cabbage
Pickled and salted vegetables	preserved vegetables, vegetables in soy sauce
Seaweed	Fresh or dried seaweed
Fruits	Fresh and canned (no added sugar) fruits
Red meat	beef, lamb, donkey, rabbit
Pork	Pork tenderloin, Pork belly, leg, rib chop
Organ meats	Liver, kidney, large intestine, blood
Processed meats	Sausages, ham, luncheon meat, dried meat, smoked meat
Poultry and game	Chicken, duck, goose
Eggs and eggs and products	Whole eggs, yolk, white, preserved eggs
Fish and seafood	Fresh- and salt-water fish, dried fish, shellfish
Milk and dairy products	Cow milk, goat milk, skim milk, flavored milk, cheese, yogurt
Fast food	Onion rings, potato chips, Western-style fast-food, salty snacks
Candy	Jelly, jam, chocolate, honey, sugar, candies
Sweetened beverages	Fruit or flavored drinks, fruit juice, soft drinks

The traditional Chinese dietary pattern was loaded heavily on rice, red meat, pork, poultry, vegetables (leafy) and fish. The modern dietary pattern was loaded heavily on wheat buns, cakes, legume products, nuts, pickled and salted vegetables, fruit, red meat, processed meats, poultry, eggs, fish, milk and fast food

### Outcome variables

Height and weight were measured by using a standardized protocol from the World Health Organization (WHO) [27] in which height is measured to the nearest 0.1 cm, as participants stand with their backs against a wall, with no shoes and with their eyes looking straight ahead. Weight was measured according to the WHO protocol, with a lever balance to the neared 0.1 kg, in participants wearing minimal undergarments and no shoes. Body mass index (BMI) was then calculated as weight (in kilograms) divided by squared height (in meters). Obesity was defined according to age and sex by using the WHO BMI growth reference [5–19] years.

### Covariates

Covariates including age, sex, residency, highest level of parental education, region, physical activity and energy intake were collected by in-home visit interviews and general information questionnaires. Residency was classified into two categories (urban and rural), region was classified into three categories (western, eastern and central), highest level of parental education was classified into five categories (illiterate or primary school; middle school; high school; technical or vocational degree; and college or higher). Physical activity was calculated as duration of total physical activity and expressed in hours per week (hours/week). Energy intake was calculated according to the China Food Composition and expressed in kilocalories per day (kcal/day).

### Statistical analysis

Dietary patterns were identified at baseline (in 2006) by factor analysis using the principal component method. The factor scores were orthogonally (varimax) rotated to create less correlation among the patterns and to facilitate interpretability. An eigenvalue is the variance of the factor. In the initial factor solution, the first factor will account for the most variance, the second will account for the next highest amount of variance, and so on. According to the previous study [16, 28], dietary patterns were identified on the basis of the eigenvalue (> 2) and scree plot. Percentages of variances were also calculated. Factor loading of >|0.20| was included to represent the food strongly associated with the identified factor [29]. Pattern-specific factor scores were calculated as the sum of the food factor loading coefficients and the standardized daily consumption of food related to the dietary pattern. Factor scores were divided into four quartiles on the basis of their contribution to each pattern, and an increase from Q1 to Q4 was assumed.

ANOVA tests for continuous variables and chi-square tests for categorical variables were used to compare the different quartiles in dietary patterns at baseline. Ordered logistic regression models were used to estimate the odds ratios (OR) and 95% confidence intervals (95%CI) for later childhood obesity across the quartile categories of dietary pattern score. Model 1 was adjusted for sex and age. Model 2 was additionally adjusted for residency, highest level of parental education, region, physical activity and energy intake. Model 3 additionally used a cluster analysis to reduce the spatial interdependence, which can adversely affect the robustness of regression estimates [30]; we used the classifications of geographical regions as cluster groups to analyze the association.

To gain access to the full sample, we used the multiple imputation technique to fill in the missing values ten times, a procedure suggested to be sufficient in a previous study [31]. Imputation was performed by using multivariate normal regression. A *p*-value < 0.05 was regarded as statistically significant. All analyses were performed in Stata (version 13.0; StataCorp, College Station, TX, USA).

## Results

Examples of food items for each food group are shown in Table 1. The baseline characteristics of participants, according to quartiles for each dietary pattern, are shown in Table 2. At baseline, 37 participants (7.57%) were overweight, while 56 participants (11.45%) had obesity. Participants in the highest quartile of the traditional dietary pattern were more likely to be significantly older, live in the central of China, had a lower prevalence of obesity and higher energy intake than those in the lowest quartile. Conversely, participants in the highest quartile of the modern dietary pattern were more likely to live in urban, eastern of China, had a higher parental education level and higher energy intake than those in the lowest quartile (*p* < 0.05).

**Table 2:** Baseline characteristics of participants according to quartiles of dietary patterns

	Traditional Chinese Dietary pattern scores					*p*-value
	All	Q1	Q2	Q3	Q4	
N	489	122	122	123	122	
Sex (%)						0.835
Boy	249 (50.92)	65 (53.28)	63 (51.64)	58 (47.54)	63 (51.22)	
Girl	240 (49.08)	57 (46.72)	59 (48.36)	64 (52.46)	60 (48.78)	
Age in years (Mean SD)	9.08 (2.76)	8.69 (2.63)	8.20 (2.69)	9.47 (2.83)	9.95 (2.58)	**< 0.001**
Residency (%)						0.075
Urban	142 (29.04)	33 (27.05)	41 (33.61)	42 (34.43)	26 (21.14)	
rural	347 (70.96)	89 (72.95)	81 (66.39)	80 (65.57)	97 (78.86)	
Highest level of parental Education (%)^a^						0.105
Illiterate or primary school	101 (20.74)	17 (13.93)	23 (19.17)	29 (23.77)	32 (26.02)	
Junior middle school	224 (46.00)	58 (47.54)	62 (51.67)	53 (43.44)	51 (41.46)	
High middle school	84 (17.25)	25 (20.49)	22 (18.33)	18 (14.75)	19 (15.45)	
Technical or vocational degree	50 (10.27)	18 (14.75)	10 (8.33)	11 (9.02)	11 (8.94)	
College or higher	28 (5.75)	4 (3.28)	3 (2.50)	11 (9.02)	10 (8.13)	
Region (%)						**< 0.001**
Western	104 (21.27)	10 (8.20)	29 (23.77)	30 (24.59)	35 (28.46)	
Eastern	147 (30.06)	59 (48.36)	45 (36.89)	33 (27.05)	10 (8.13)	
Central	238 (48.67)	53 (43.44)	48 (39.34)	59 (48.36)	78 (63.41)	
Obesity status (%)						**0.01**
Non-overweight or obesity	396 (80.98)	84 (68.85)	102 (83.61)	107 (87.70)	103 (83.74)	
Overweight	37 (7.57)	15 (12.30)	7 (5.74)	7 (5.74)	8 (6.50)	
Obesity	56 (11.45)	23 (18.85)	13 (10.66)	8 (6.56)	12 (9.76)	
Physical activity (Mean SD)	7.06 (5.32)	6.67 (5.17)	7.28 (5.22)	7.39 (5.51)	7.03 (5.54)	0.265
Energy (Mean SD)^b^	1462.43 (530.01)	1285.73 (455.83)	1243.43 (415.33)	1454.80 (495.97)	1862.476 (511.79)	**< 0.001**
	Modern Dietary pattern scores					
	All	Q1	Q2	Q3	Q4	*p*-value
N	489	122	122	122	123	
Sex (%)						0.204
Boy	249 (50.92)	65 (53.28)	52 (42.62)	67 (54.92)	65 (52.85)	
Girl	240 (49.08)	57 (46.72)	70 (57.38)	55 (45.08)	58 (47.15)	
Age in years (Mean SD)	9.08 (2.76)	8.80 (2.63)	9.16 (2.79)	9.11 (2.95)	9.24 (2.67)	0.6044
Residency (%)						**< 0.001**
Urban	142 (29.04)	25 (20.49)	28 (22.95)	35 (28.69)	54 (43.90)	
rural	347 (70.96)	97 (79.51)	94 (77.05)	87 (71.31)	69 (56.10)	
Highest level of parental Education (%)^a^						**< 0.001**
Illiterate or primary school	101 (20.74)	39 (32.50)	36 (29.50)	11 (9.02)	15 (12.20)	
Junior middle school	224 (46.00)	59 (49.17)	58 (47.53)	61 (50.00)	46 (37.40)	
High middle school	84 (17.25)	17 (14.17)	19 (15.57)	22 (18.03)	26 (21.14)	
Technical or vocational degree	50 (10.27)	3 (2.50)	7 (5.74)	19 (15.57)	21 (17.07)	
College or higher	28 (5.75)	2 (1.67)	2 (1.64)	9 (7.38)	15 (12.20)	
Region (%)						**< 0.001**
Western	104 (21.27)	60 (49.18)	32 (26.23)	7 (5.74)	5 (4.07)	
Eastern	147 (30.06)	10 (8.20)	20 (16.39)	39 (31.97)	78 (63.41)	
Central	238 (48.67)	52 (42.62)	70 (57.38)	76 (62.3)	40 (32.52)	
Obesity status (%)						0.208
Non-overweight or obesity	396 (80.98)	108 (88.52)	99 (81.15)	94 (77.05)	95 (77.24)	
Overweight	37 (7.57)	3 (2.46)	10 (8.20)	12 (9.84)	12 (9.76)	
Obesity	56 (11.45)	11 (9.02)	13 (10.66)	16 (13.11)	16 (13.01)	
Physical activity (Mean SD)	7.06 (5.32)	7.33 (5.78)	6.90 (5.39)	7.02 (5.18)	7.01 (5.03)	0.797
Energy (Mean SD)^b^	1462.43 (530.01)	1325.28 (461.8118)	1386.38 (488.41)	1461.66 (552.026)	1674.66 (550.73)	**< 0.001**

*p*-value was calculated from χ^2^ test for binary variables or by ANOVA for continuous variables. Dietary patterns were identified at baseline (in 2006). *p*-values< 0.05 are bold. ^a^: *n* = 487 cause of the missing data. ^b^: *n* = 435 cause of the missing data

Two dietary patterns were determined in the present study. The factor loading of each pattern is shown in Table 3. The traditional Chinese dietary pattern (Eigenvalue = 2.36) was loaded heavily on rice, red meat, pork, poultry, vegetables (leafy) and fish, and was inversely loaded on wheat flour, wheat buns, vegetables (non-leafy), corn and eggs. The modern dietary pattern (Eigenvalue = 2.22) was loaded heavily on wheat buns, cakes, legume products, nuts, pickled and salted vegetables, fruit, red meat, processed meats, poultry, eggs, fish, milk and fast food, and was inversely loaded on wheat noodles. The two patterns together explained 16.36% of the dietary intake variance.

**Table 3:** Factor loadings for dietary patterns derived from exploratory factor analysis

	Traditional pattern	Modern pattern
Rice	0.7401	
Wheat noodles		−0.2918
Wheat flour	−0.4377	
Wheat buns, breads	−0.5472	0.2077
Cakes, cookies and pastries		0.4780
Deep-fried wheat		
Corn and coarse grain	−0.3337	
Starchy roots and tubers		
Fresh legumes		
Dried legumes		
Legume products		0.3734
Nuts and seeds		0.4711
Fresh vegetables, non-leafy	−0.2290	
Fresh vegetables, leafy	0.6119	
Pickled and salted vegetables		0.2529
Seaweed		
Fruits		0.5154
Red meat	0.2667	0.2245
Pork	0.4855	
Organ meats		
Processed meats		0.3149
Poultry and game	0.2555	0.3335
Eggs and eggs products	−0.3168	0.3282
Fish and seafood	0.2524	0.4346
Milk and dairy products		0.4770
Fast food		0.4726
Candy		
Sweetened beverages		

Dietary patterns were identified at baseline (in 2006). Absolute values< 0.20 are excluded from the table for simplicity

The relationship between dietary patterns and later obesity in each wave, as assessed with the ordered logistic model, is displayed in Tables 4 and 5, respectively. In Table 4, dietary patterns were identified at baseline (in 2006), whereas participants’ obesity status was measured in 2009. In 2009, after cluster analysis and adjustment for confounders, children in the highest quartile (Q4) of the traditional Chinese dietary pattern had lower odds of obesity (OR = 0.29, 95%CI: 0.16, 0.51 for Q4) than those in the lowest (Q1), whereas children in Q4, Q3 and Q2 of the modern dietary pattern had greater odds of obesity than those in Q1 (OR = 3.22, 95%CI: 1.66, 6.23 for Q4; OR = 2.46, 95%CI: 2.17, 2.80 for Q3; OR = 2.22, 95%CI: 1.42, 3.46 for Q2). Moreover, in Table 5, participants’ obesity status were measured in 2011, children in Q4 and Q3 of the traditional Chinese dietary pattern still had lower odds of obesity (OR = 0.19, 95%CI: 0.09, 0.40 for Q4; OR = 0.47, 95%CI: 0.33, 0.67 for Q3) than those in Q1, whereas children in Q4 of the modern dietary pattern still had greater odds of obesity (OR = 2.02, 95%CI: 1.17, 3.48) than those in Q1.

**Table 4:** Multivariable adjusted ORs and 95%CI for obesity in 2009 across quartiles of dietary pattern scores

	Traditional Chinese Dietary pattern				
	Q1	Q2	Q3	Q4	P for trend
Model1	1	0.87 (0.43, 1.75)	0.60 (0.29, 1.28)	**0.34 (0.14, 0.82)**	**0.010**
Model2	1	1.04 (0.52, 2.10)	0.66 (0.31, 1.41)	**0.29 (0.10, 0.78)**	**0.015**
Model3	1	1.04 (0.54, 2.00)	0.66 (0.31, 1.40)	**0.29 (0.16, 0.51)**	**0.002**
	Modern Dietary Pattern				
	Q1	Q2	Q3	Q4	P for trend
Model1	1	2.09 (0.84, 5.20)	**2.47 (1.01, 6.07)**	**3.18 (1.33, 7.64)**	**0.009**
Model2	1	2.22 (0.87, 5.63)	2.46 (0.99, 6.14)	**3.22 (1.26, 8.18)**	**0.018**
Model3	1	**2.22 (1.42, 3.46)**	**2.46 (2.17, 2.80)**	**3.22 (1.66, 6.23)**	**0.006**

Model 1: adjusted for sex and age. Model 2: additionally adjusted for residency, highest level of parental education, region, physical activity and energy intake. Model 3: additionally included use of classifications of geographical regions as cluster groups to analyze the association. Dietary patterns were identified at baseline (in 2006). *p*-value for trend was obtained by adjusting the quartiles of the pattern scores as a continuous variable. *p*-values< 0.05 are bold

**Table 5:** Multivariable adjusted ORs and 95%CI for obesity in 2011 across quartiles of dietary pattern scores

	Traditional Chinese Dietary Pattern				
	Q1	Q2	Q3	Q4	P for trend
Model1	1	1.00 (0.52, 1.95)	0.52 (0.24, 1.12)	**0.28 (0.11, 0.70)**	**0.002**
Model2	1	1.06 (0.54, 2.08)	0.47 (0.21, 1.02)	**0.19 (0.07 0.49)**	**< 0.001**
Model3	1	1.06 (0.30, 3.69)	**0.47 (0.33, 0.67)**	**0.19 (0.09, 0.40)**	**< 0.001**
	Modern Dietary Pattern				
	Q1	Q2	Q3	Q4	P for trend
Model1	1	1.30 (0.56, 3.06)	1.67 (0.75, 3.77)	2.17 (0.99, 4.77)	**0.039**
Model2	1	1.28 (0.54, 3.01)	1.58 (0.69, 3.65)	2.02 (0.88, 4.65)	0.086
Model3	1	1.28 (0.48, 3.44)	1.58 (0.74, 3.39)	**2.02 (1.17, 3.48)**	0.078

Model 1: adjusted for sex and age. Model 2: additionally adjusted for residency, highest level of parental education, region, physical activity and energy intake. Model 3: additionally included use of classifications of geographical regions as cluster groups to analyze the association. Dietary patterns were identified at baseline (in 2006). *p*-value for trend was obtained by adjusting the quartiles of the pattern scores as a continuous variable. *p*-values< 0.05 are bold

## Discussion

In the present study, we report analysis of dietary patterns among Chinese children aged 6–14 at baseline and the association of dietary patterns with later obesity. Two dietary patterns were identified in the multicenter longitudinal study that satisfactorily captured eating habits (16.36% of dietary intake variance explained). The first pattern, the traditional Chinese dietary pattern, was characterized mainly by high consumption of rice, vegetables, pork, red meat, poultry and fish. The second pattern, the modern dietary pattern, consisted of a combination of wheat buns, bread, cake, cookies, legumes, pickled (salted) vegetables, fruits, nuts, red meat, processed meat, poultry, eggs, fish, milk and fast food. The identified “traditional” and “modern” dietary patterns are similar to those reported previously conducted among adults in China [16, 17, 21].

In the present study, the traditional Chinese dietary pattern was inversely associated with later obesity, whereas the modern dietary pattern was positively associated with later obesity. Our results are comparable to those of previous studies. The Dutch Lifelines cohort study indicated that the healthier dietary pattern which have high intake of fruit, vegetables and fish were inversely associated with obesity, whereas the modern dietary pattern which have high intake of bread, potatoes and sweet snacks had a significantly increased risk of obesity [32]. In a 6-year prospective study conducted in UK, Ambrosini et al. [33] also reported that a dietary pattern high in meat, energy density and fat might be positively associated with obesity. However, previous studies have mainly focused on adults [22, 23] and cross-sectional designed [16, 21]. Some findings relating dietary patterns and obesity may not be applicable to Chinese adolescents and children, owing to cultural factors affecting intake.

Overall, the present results suggested that the traditional Chinese dietary pattern may be protective against later obesity. The protective effect may be attributed to its healthful components. For example, the benefit of consuming whole grains, vegetables and legumes as part of the traditional Chinese dietary pattern may explain the protective effect in our study [16, 22]. First, rice, an essential staple food in China, is a low-energy food that constitutes the bulk of the traditional Chinese diet. Energy density of rice is lower than that of wheat [15]. Different methods are used in cooking rice and wheat. Steamed rice contains twice the amount of water and half the energy content as an equivalent amount of bread [22]. Second, instead of organ and processed meats, the traditional Chinese dietary pattern includes a high intake of white meat, fish and seafood, which are all associated with a low prevalence of obesity. Fish has been found to be protective against obesity because of its omega-3 polyunsaturated fatty acid content [34]. White meat has less fat than red meat, and the fats present are primarily healthful unsaturated fats [31, 35]. Another potential explanation for our findings may be vegetable intake. Vegetables that provide large amounts of dietary fiber, antioxidants (e.g., vitamins C and E) and water may contribute to decreasing the risk of later obesity [16, 36]. However, in the present study, fresh leafy and non-leafy vegetables showed opposite effects in factor loadings. We assumed that the different cooking methods using in preparing vegetables might explain the opposite effects. For instance, stir-frying non-leafy vegetables in vegetable oil may increase energy density [22, 37].

The present study also found a higher risk of obesity associated with the modern dietary pattern. Previous studies have shown positive associations between modern dietary patterns abundant in saturated fat and cholesterol [16, 38, 39] and a higher risk of obesity. The associations between the modern dietary pattern and later obesity may be attributed to the high amounts of suboptimal foods together with the lower fiber intake [29, 33, 40]. Wheat gluten may promote weight gain, partly by decreasing the thermogenic capacity of adipose tissue [21]. Second, high consumption of red meat and processed meat, containing amounts of saturated fat and cholesterol, is directly associated with an increased risk of obesity [16, 35, 41]. Another characteristic of the modern pattern is the use of the unhealthful cooking method deep-frying [37]. Consumption of deep-fried fast food, such as potato chips and deep-fried dough, is of serious concern. A prospective cohort study has suggested that greater consumption of fried food is associated with a higher risk of obesity in follow-up [42]. Additionally, in this study, we found that milk and dairy products were positively associated with the modern pattern score. A previous study has suggested that milk intake associated with risk of childhood obesity, and the insulinotropic and IGF-1-raising effects of milk and dairy products may have further adverse effects on obesity [43]. Moreover, excess energy intake may play a role in the development of obesity [44]. After adjustment for energy intake, we found that the associations for each dietary pattern remained significant.

To our knowledge, this is the first study reporting the relations between dietary patterns and later obesity risk among the children and adolescents in China. Our findings provided valuable information for the primary prevention of childhood obesity through the dietary modifications in a Chinese children and adolescents. However, it is important to note some potential limitations. Firstly, the present study was limited by old data source and small sample sizes (here we report 489 participants) which may not necessarily representative of the general Chinese populations. Second, there was a high rate of loss of subjects to follow-up because of migration and city construction. However, there was no significant difference between those lost in follow-up and those retained. Thus, the association between dietary patterns and the risk of later obesity might not have been biased. Thirdly, dietary data were collected through three 24-h dietary recalls, which might not represent long-term usual intake, and using a baseline dietary data might provide a biased estimate. Fourthly, some subjective and arbitrary decisions should be considered, including the number of factors retained and the labeling of dietary patterns. However, these aspects are common to factor analyses, and the methodology has been validated and found satisfactory [22]. Finally, even though we controlled for many covariates, there are still some potential socioeconomic and genetic factors that may have confounded our estimation.

## Conclusion

In conclusion, the present study identified two dietary patterns among Chinese adolescents and children aged 6–14 years at baseline: the traditional Chinese pattern and the modern pattern. The traditional Chinese dietary pattern was inversely associated with later obesity, whereas the modern dietary pattern was positively associated with later obesity. These findings further confirmed the importance of dietary patterns among children in later obesity and lay a groundwork for culture-specific interventions targeted at reducing rates of childhood obesity. Interventions aiming at discouraging the adoption of the modern dietary pattern, such as consumption of fried food, processed meat, and energy-dense foods, while encouraging the traditional Chinese dietary pattern, should be developed.
